# Retrograde visceral revascularization using iliac branch stent graft for postdissection thoracoabdominal aortic aneurysm

**DOI:** 10.1016/j.jvscit.2025.101955

**Published:** 2025-08-21

**Authors:** Xiaojun Shu, Yonglong Wang, Dan Zhou, Weijian Chen, Zhengfei Li, Yingxin Tan

**Affiliations:** a Department of Vascular Surgery, General Surgery, The First Hospital of Lanzhou University, Lanzhou, China; b The First School of Clinical Medicine, Lanzhou University, Lanzhou, China; c Heart Center, The First Hospital of Lanzhou University, Lanzhou, China; d School of Clinical Medicine, Tsinghua University, Beijing, China

**Keywords:** Post-dissection thoracoabdominal aortic aneurysm, Retrograde visceral revascularization, Stent graft

## Abstract

This case report describes a novel endovascular approach for retrograde visceral revascularization using an inverted iliac branch stent graft in a high-risk patient with a chronic postdissection thoracoabdominal aortic aneurysm. Through an 8.9-mm entry tear in the left common iliac artery communicating with the false lumen, a premanufactured iliac branch stent graft was deployed retrograde to reconstruct the celiac trunk and left renal artery. This technique was combined with physician-modified fenestrated endografting for antegrade superior mesenteric and right renal artery revascularization. Complete aneurysm exclusion and branch preservation were achieved without open surgery, with patency confirmed at the 1-year follow-up. This strategy offers an alternative for anatomy challenging standard fenestrated/branched endografts.

Chronic postdissection thoracoabdominal aneurysms exhibit rapid progression, with 63% demonstrating aneurysmal degeneration within 5 years despite initial endovascular management (proximal thoracic endovascular aortic repair without distal false lumen intervention), necessitating innovative solutions for high-risk patients.^1^ This case uses a retrograde visceral reconstruction technique with a premanufactured iliac branch stent graft system—a novel technique circumventing open debranching—leveraging the technical feasibility demonstrated in contemporary physician-modified endograft (PMEG) series for complex anatomy.^2^^,^^3^ The patient provided consent for this case report.

## Case report

**Fig 1:**
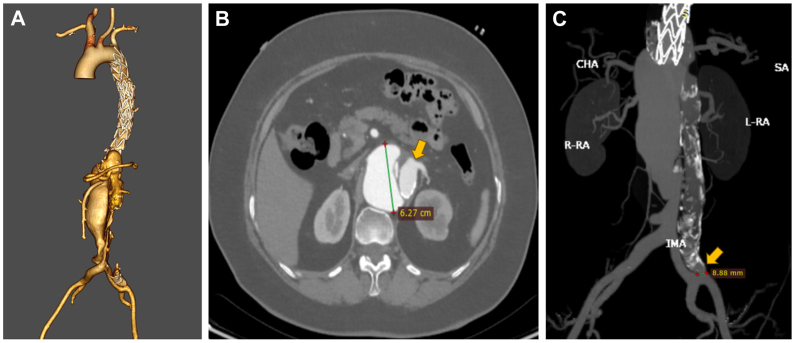
**(A)** Computed tomography angiography (CTA) revealed a postdissection thoracoabdominal aortic aneurysm, with the celiac trunk and left renal artery arising entirely from the false lumen. **(B)** CTA demonstrated a postdissection thoracoabdominal aortic aneurysm measuring 62.7 mm in diameter. The *arrow* indicates the left renal artery arising entirely from the false lumen. **(C)** CTA demonstrates an 8.9-mm entry tear in the left common iliac artery communicating with the false lumen (*arrow*).

A 79-year-old woman with hypertension and a history of thoracic endovascular aortic repair performed 4 years prior at an outside institution (specific device details unavailable) presented with a 2-week history of intermittent abdominal pain. Preoperative computed tomography angiography demonstrated a postdissection thoracoabdominal aortic aneurysm measuring 62.7 mm in diameter (Fig 1, *A*). Notably, the celiac trunk and left renal artery arose entirely from the false lumen (Fig 1, *B*). An 8.9-mm tear in the left common iliac artery communicated with the false lumen (Fig 1, *C*). Given her advanced age and refusal of open surgery after counseling, a total endovascular approach was pursued. The strategy employed a hybrid PMEG with selective fenestration for the superior mesenteric artery (SMA) and right renal artery, combined with retrograde branched graft reconstruction for the celiac trunk and left renal artery. This approach was selected for three key reasons^1^: optimal anatomical conditions—the SMA and right renal artery originated from the true lumen, enabling precise fenestration alignment^2^; technical feasibility—limiting to two fenestrations reduced procedural complexity^3^; prohibitive false lumen challenges—the celiac trunk and left renal artery arose entirely from the false lumen with fibrotic septal thickening, rendering septal puncture and antegrade alignment unfeasible.

**Fig 2:**
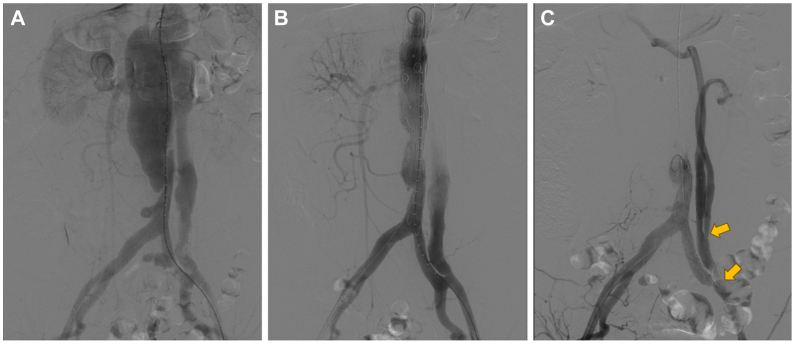
**(A)** Arteriography demonstrated nonopacification of the left renal artery and its branches. **(B)** The superior mesenteric artery (SMA) and right renal artery were successfully reconstructed using a physician-modified endograft (PMEG) technique. **(C)** Retrograde reconstruction of the celiac trunk and left renal artery using a premanufactured iliac branch stent graft and covered stents. The *arrow* indicates the original iliac artery stent's internal iliac artery opening.

After induction of general anesthesia, bilateral percutaneous common femoral artery access was obtained. Diagnostic angiography revealed a dissecting abdominal aortic aneurysm (Fig 2, *A*). A physician-modified fenestrated endograft (Lifetech Scientific) with two reinforced fenestrations targeting the SMA and right renal artery ostia was deployed via right femoral access to reconstruct target vessels and exclude the proximal entry tear. Both vessels were reconstructed with two 8-mm Viabahn stent grafts (W. L. Gore & Associates) (Fig 2, *B*).

The premanufactured iliac branch stent graft (G-iliac; IB-1412-050-120; Lifetech Scientific) was removed from its 18F delivery sheath, rotated 180°, and reloaded for retrograde deployment. Via left common femoral artery access, the device was advanced with its branch segment oriented cephalad. Our strategy was to land the common iliac body of the G-iliac device in the patient's left external iliac artery, the external iliac branch of the G-iliac device in the patient's left common iliac artery and to position the internal iliac branch of the G-iliac device retrograde into the false lumen via the tear. A secondary reversed iliac branch stent graft (G-iliac; IB-1412-050-120; Lifetech Scientific) was deployed through the internal iliac artery portal of this primary stent graft. Via the secondary iliac branch stent graft, selective cannulation of the celiac trunk and left renal artery was achieved using a 0.035-inch guidewire (Abbott Vascular), followed by reconstruction of both vessels with Viabahn stent grafts (W. L. Gore & Associates). Final angiography confirmed effective retrograde perfusion of both the left renal artery and celiac trunk (Fig 2, *C*).

**Fig 3:**
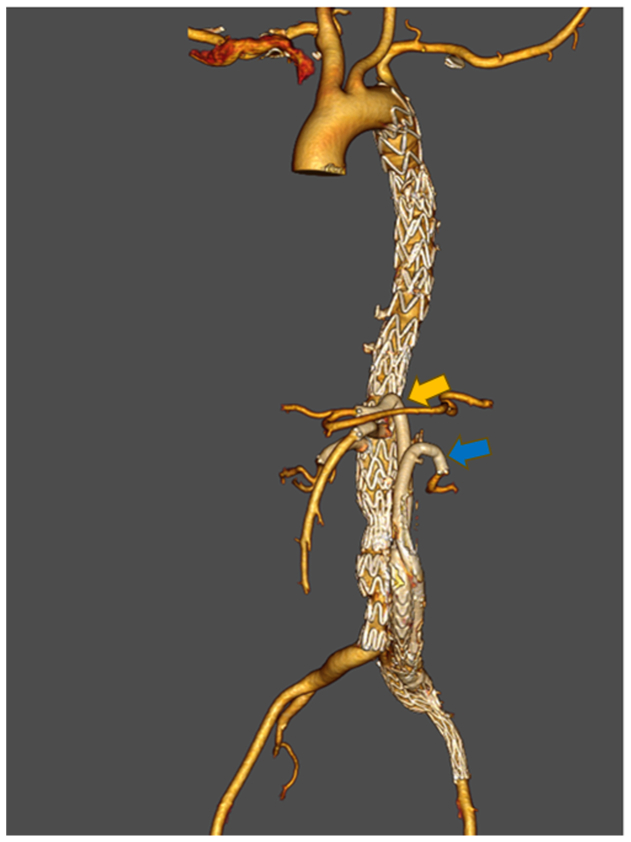
One-year follow-up computed tomography angiography demonstrating complete false lumen thrombosis. *Arrows* indicate patent Viabahn stent grafts in the retrograde-reconstructed celiac trunk (*yellow*) and left renal artery (*blue*).

The patient was discharged after 7 days without complications (eg, spinal cord ischemia, renal/liver dysfunction). One-year follow-up demonstrated complete false lumen thrombosis and patent reconstructed vessels (Fig 3).

## Discussion

This report details a novel endovascular strategy for managing a postdissection thoracoabdominal aneurysm. The approach integrates three key innovations: retrograde visceral revascularization via a premanufactured iliac branch stent graft, physician-modified fenestration for antegrade branch reconstruction, and total endovascular exclusion, thus avoiding open surgery.

The use of an iliac branch stent graft for retrograde celiac trunk and left renal artery reconstruction circumvented the need for complex fenestrations in a hostile aortic anatomy. The technique exploits the tear in the iliac artery as a stable inflow source, significantly simplifying the procedure compared with antegrade fenestration. Similar retrograde revascularization principles were historically achieved via open iliac-visceral bypasses,^4^ but the endovascular adaptation eliminates laparotomy-related morbidity. The premanufactured iliac branch system offered standardized component alignment and deployment stability, reducing procedural time compared with PMEGs.^2^^,^^3^

Chronic postdissection thoracoabdominal aneurysms often feature some branch vessels arising entirely from the false lumen, complicating conventional endovascular repair.^1^^,^^5^ In this case, the celiac trunk and left renal artery arose from the false lumen. We performed retrograde visceral artery reconstruction via the iliac branch stent graft within the false lumen, thus offering an alternative solution for patients where some visceral arteries arise entirely from the false lumen and present alignment challenges for antegrade fenestration techniques. Chen et al^5^ reported significantly higher endoleak rates for target vessels originating from the FL after fenestrated/branched endovascular aneurysm repair (19% vs 4%; *P* < .001).

## Conclusions

This novel hybrid endovascular approach combines retrograde iliac branch stent grafting via an iliac tear with physician-modified fenestration to reconstruct visceral arteries arising from the false lumen in a high-risk chronic postdissection thoracoabdominal aneurysm. It achieved complete exclusion and branch preservation without open surgery, demonstrating 1-year patency and offers an alternative for challenging anatomy that makes using a standard fenestrated/branched endovascular aneurysm repair difficult. Future studies should validate the scalability of this technique in larger cohorts.

## Ethics approval and consent to participate

The patient provided written informed consent for publication. This study was approved by the Ethics Committee of The First Hospital of Lanzhou University [LDYYLL2024-245].

## Funding

These authors funded by Gansu Province Education Technology Innovation Project No.2022B-009, the fund of the First Hospital of 10.13039/100012899Lanzhou University Fund-ldyyyn2021-64, Young Scientists' Fund of the 10.13039/501100009620Gansu Provincial Science and Technology Program No.23JRRA1615 and Excellent Doctoral Program of 10.13039/501100009620Gansu Provincial Science and Technology Program No.22JR5RA890 and The 10.13039/501100009620Gansu Provincial Science and Technology Program No.21JR7RA358.

## Disclosures

None.
